# Staging Laparoscopy in Carcinoma of Stomach: A Comparison with CECT Staging

**DOI:** 10.1155/2013/674965

**Published:** 2013-05-02

**Authors:** Showkat Majeed Kakroo, Arshad Rashid, Ajaz Ahmad Wani, Zahida Akhtar, Manzoor Ahamad Chalkoo, Asim Rafiq Laharwal

**Affiliations:** ^1^Department of General Surgery, Government Medical College, Srinagar 190010, India; ^2^Minimal Access Surgery, Lok Nayak Hospital, Maulana Azad Medical College, New Delhi 110002, India

## Abstract

*Background*. aim of this study was to compare the role of diagnostic laparoscopy and contrast enhanced computed tomography (CECT) of abdomen in the staging of stomach carcinoma. *Methods*. This was a prospective study conducted in a tertiary care hospital over a period of two years and included 50 patients of endoscopy and biopsy proven stomach carcinoma that were found to be operable on CECT. Diagnostic laparoscopy was performed in all patients before proceeding to a formal laparotomy. *Results*. Metastasis was detected at diagnostic laparoscopy in 14 (28%) patients. CECT correctly identified the T stage in 22 (61%) patients. Overall accuracy of CECT for T staging was 74% with a a sensitivity of 65% and a specificity of 79%. Laparoscopy correctly identified the T stage in 26 (72%) patients. Overall accuracy of laparoscopy for T staging was 81% with a sensitivity of 76% and specificity of 86%. the most common N stage on CECT was N0 (50%). CECT correctly identified the N stage in 26 (72%) patients. Overall accuracy of CECT for N staging was 86% with a sensitivity of 50% and a specificity of 90%. the most common N stage on laparoscopy was N0 and N2 (42% each). Laparoscopy correctly identified the N stage in 27 (75%) patients. Overall accuracy of Laparoscopy for N staging was 88% with a sensitivity of 53% and specificity of 91%. *Conclusion*. Laparoscopy is a valuable technique in staging of stomach carcinoma and has an important role in the detection of intra-abdominal metastasis missed by CECT.

## 1. Introduction

Gastric cancer remains one of the most common causes of death from cancer worldwide, especially in our part of the world. In Kashmir, the incidence rates for gastric cancer have been estimated at 36.7/100000 per year in men and 9.9/100000 per annum in women, respectively [1]. As the multidisciplinary management of gastrointestinal cancer has evolved over the last decade, an accurate extent of disease workup has become essential for treatment planning. Even after a thorough radiological workup, many patients with stomach carcinoma are diagnosed as unresectable or metastatic on exploratory laparotomy. For the subgroup of patients who do not require palliation, exploration confers little benefit and may, on the contrary, be associated with significant morbidity and mortality [2]. Since the introduction of contrast enhanced computed tomography (CECT) scan some 30 years back, the staging workup of gastric carcinoma has underwent a boom [3–5]. CECT is used preoperatively primarily to determine the stage and extragastric spread of the carcinoma but has the propensity to underestimate the extent of disease, with small-volume metastatic disease being appreciated only at open surgical exploration. Laparoscopy has been suggested as a means for identifying such small-volume disease. The aim of laparoscopic staging is to mimic staging at open exploration while minimizing morbidity, enhancing recovery, and thus allowing for quicker administration of adjuvant therapies if indicated [6–8].

## 2. Methods and Materials**

This was a prospective study conducted on 50 patients of endoscopic and biopsy proven stomach carcinoma that were found to be operable on CECT of abdomen/pelvis. The study was conducted over a period of two years in a tertiary care hospital of Kashmir. All the patients were staged preoperatively by CECT of abdomen/pelvis done on a 32-slice helical CT scanner (Fxi, GE Medical Systems). Patients were kept fasting for six hours prior to their scan. The patients were asked to take 500 mL, 250 mL, and 25 mL of water orally 120, 60, and 5 minutes, respectively, prior to their scan. Five mm contiguous cuts were taken from the dome of diaphragm to the pubic rami. Scans were taken after intravenous administration of 100 mL 60% iodinated contrast agent. Any area of gastric wall with thickness measuring more than 5 mm was considered abnormal. Irregularities in the external surface of wall were considered serosal involvement. Tumors confined to the gastric wall or intramural or transmural involvement with a smooth outer wall and clear fat plane around tumor were considered T1/T2. Transmural tumors with irregular or blurred outer border with or without perigastric fat stranding were considered as T3. Obliteration of fat plane between gastric tumor and adjacent organ or direct invasion of adjacent organ was taken as T4. Any enlarged lymph node seen in the 16 anatomic sites as per the Japanese Research Society on Gastric Cancer classification was noted as nodal disease [9]. Regional lymph nodes were considered to represent local metastases if they were solitary or separate nodes 8 mm or greater in long-axis diameter with enhancement, which was defined as attenuation greater than 85 Hounsfield units in the postcontrast portal venous phase. The CECT films were fully reviewed and discussed with a qualified radiologist.

Diagnostic laparoscopy was done in all these patients before proceeding with a formal exploratory laparotomy. This procedure was explained to the patients/attendants in detail and an informed consent was taken for the same. Closed technique was used to gain access into abdomen. A formal diagnostic laparoscopy was undertaken through a subumbilical port. After a thorough inspection of all four quadrants of the peritoneal cavity was carried out, biopsies were taken from any suspicious tissue. The lesser sac was inspected routinely and accessory ports were employed if needed. Peritoneal lavage was not included in the diagnostic laparoscopy protocol. Definitive surgery was performed on the patients who were found resectable on laparoscopy.

A formal staging of the patient was done as per the 7th edition of the UICC/TNM Classification [10], and a comparison between the staging obtained from CECT and that from laparoscopy was made. Statistical Analysis was done by *Graphpad Instat Version 3.10 *for Windows (Graphpad softwares Inc., San Diego, CA, USA). An ethical clearance was obtained from the local ethics committee.

## 3. Results

Fifty consecutive patients of stomach carcinoma, found to be resectable on CECT, were enrolled. The mean age of presentation was 58.57 ± 5.7 years in males and 56.67 ± 6.3 years in females. The maximum incidence of stomach carcinoma in our study was found in the age group of 56 to 65 years. Males outnumbered females by a factor of 2.85 : 1.

Metastasis was detected at diagnostic laparoscopy in 14 (28%) patients. Hepatic metastasis was the most common (9 patients). Peritoneal metastases were seen in 5 patients either isolated (3 patients) or in association with liver metastases (2 patients) (Table 1). As these peritoneal deposits were not picked up by the CECT, comparison with diagnostic laparoscopy and histopathology was not possible, so these patients were excluded from the study and received palliative treatment. Staging with preoperative CECT was compared with the laparoscopic staging in the other 36 patients taking histopathological staging as the standard. The most common T stage on CECT was T3 and T4 (44.44% each). Overall accuracy of CECT for T staging was 74% with a sensitivity of 65% and a specificity of 79%. The most common T stage on laparoscopy was T3 (50%). Overall accuracy of Laparoscopy for T staging was 81% with a sensitivity of 76% and a specificity of 86% (Table 2). The most common N stage on CECT was N0 (50%). Overall accuracy of CECT for N staging was 86% with a sensitivity of 50% and a specificity of 90%. The most common N stage on laparoscopy was N0 and N2 (42% each). Overall accuracy of Laparoscopy for N staging was 88% with a sensitivity of 53% and a specificity of 91% (Table 3).

## 4. Discussion

**Figure 1:**
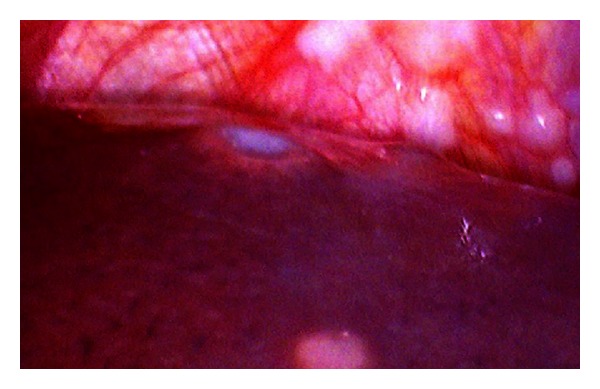
Diagnostic laparoscopy showing liver metastasis.

**Figure 2:**
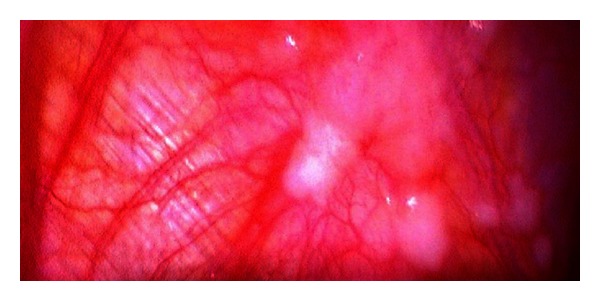
Diagnostic laparoscopy showing peritoneal metastasis on diaphragm.

In our study 50 patients underwent a diagnostic laparoscopy after a preoperative CECT excluded any form of metastasis. At diagnostic laparoscopy, out of these 50 patients, 14 patients revealed metastasis (9 hepatic, 5 peritoneal), confirmed by frozen section. Of note was one patient in whom multiple large metastases were detected on laparoscopy (Figure 1). Thus an unnecessary laparotomy was averted in 14 (28%) patients. Similar observations were made by Lowy et al. (23%), Conlon (33.7%), Sotiropoulos et al. (31.1%), and Burke et al. (37%) [11–14]. The magnification afforded by laparoscopy makes it possible to even pick up small peritoneal nodules which are otherwise missed on imaging modalities (Figure 2).

Owing to their hypervascularity, most gastric cancers are seen as enhancing lesions [15]. As regards the tumour (T) status, CECT correctly staged 22 (61%) patients. CECT over-staged 7 (19.4%) patients, and also under-staged the same number of patients. CECT had a sensitivity of 65% and a specificity of 79% for T staging. Diagnostic laparoscopy correctly staged the T status in 26 (72%) patients and it overstaged 4 (11.11%) patients, and understaged 6 patients (16.7%). Overall accuracy for T stage with laparoscopy was 81% as against 74% of CECT with a sensitivity of 76% and a specificity of 86%  (*P* = 0.0324). Our results are similar to those of the study conducted by Blackshaw et al. and D'Ugo et al. [16, 17].

As regards the nodal (N) status, CECT correctly staged 26 (72%) patients. It overstaged 4 (11.11%) patients, and understaged 6 (16.7%) patients. CECT had a sensitivity of 50% and a specificity of 90% for N staging. The relative insensitivity of CECT for detecting nodal disease is due to its inability to detect micrometastasis in the nodes [18]. Laparoscopy correctly staged N status in 27 (75%) patients, over-stage 5 (13.9%) patients, and under-stage 4 (11.11%) patients. The overall accuracy of laparoscopy for N staging was 88% as against 86% of CECT scanning with a sensitivity of 53% and a specificity of 91%  (*P* = 0.4324). Possik et al. reported an overall accuracy of laparoscopy for N staging as 58.4% with a sensitivity of 60% and a specificity of 90% [19]. Similar results were observed by a study conducted by Muntean et al. in which the overall laparoscopic N staging accuracy was 64.3% with a sensitivity of 54.5% and a specificity of 100% [20].

Laparoscopic gastrojejunostomy has been established as a safe alternative to open approach for the palliation of symptoms due to gastric outlet obstruction in unresectable cancer stomach. Additional benefits of the laparoscopic approach include decreased immune suppression, decreased postoperative pain, early ambulation, and other advantages of minimally invasive surgery [21]. However, laparoscopic gastrojejunostomy was not offered to any of our patients, as we were not adequately experienced with this procedure.

We acknowledge the fact that though the accuracy for nodal status was marginally better for laparoscopy and did not reach statistical significance, it does not preclude the use of diagnostic laparoscopy. The specific value of diagnostic laparoscopy is in detecting minimal metastatic disease that is otherwise undetectable by routine imaging modalities.

## 5. Conclusion

Laparoscopy is a valuable technique in staging stomach carcinoma and has an important role in the detection of occult extensive intra-abdominal or metastatic disease not detected by conventional radiological staging. The value of diagnostic laparoscopy is in the prevention of unnecessary surgical exploration and the resultant morbidity and mortality in patients with locally advanced or metastatic disease.

## Figures and Tables

**Table 1:** Metastases detected by laparoscopy.

Metastases	<0.5 cm	0.5–1 cm	>1 cm
Liver	6	2	1
Peritoneal	3	0	0
Both of these	0	1	1

Overall	9	3	2

**Table:** (a)

CECT T stage	Histopathologic T stage			Total	Laparoscopic T stage	Histopathologic T stage			Total
	T1/T2	T3	T4			T1/T2	T3	T4	
T1/T2	3	1	0	4	T1/T2	4	1	0	5
T3	2	8	6	16	T3	3	10	5	18
T4	2	3	11	16	T4	0	1	12	13

Total	7	12	17	36	Total	7	12	17	36

**Table:** (b)

CECT N stage	Histopathologic N stage				Total	Laparoscopic N stage	Histopathologic N stage				Total
	N0	N1	N2	N3			N0	N1	N2	N3	
N0	14	2	2	0	18	N0	12	2	1	0	15
N1	0	2	2	0	4	N1	2	3	1	0	6
N2	2	2	10	0	14	N2	2	1	12	0	15
N3	0	0	0	0	0	N3	0	0	0	0	0

Total	16	6	14	0	36	Total	16	6	14	0	36

**Table 3:** Statistical analysis of CECT and laparoscopic vis-a-vis histopathologic T and N staging.

	Sensitivity		Specifity		PPV		NPV		Accuracy	
	CT	LAP	CT	LAP	CT	LAP	CT	LAP	CT	LAP
T status										
T1/T2	75	80	88	90	43	57	97	97	86	89
T3	50	56	80	89	67	83	67	67	67	72
T4	69	92	70	78	65	71	74	95	69	83

Overall	65	76	79	86	58	70	79	86	74	81

N status										
N0	78	80	89	81	88	75	80	85	83	81
N1	50	50	88	90	33	50	94	90	83	83
N2	71	80	82	90	71	86	82	86	78	86
N3	0	0	100	100	0	0	100	100	100	100

Overall	50	53	90	91	48	53	89	90	86	88
